# Using a Mystery-Caller Approach to Examine Access to Prostate Cancer Care in Philadelphia

**DOI:** 10.1371/journal.pone.0164411

**Published:** 2016-10-10

**Authors:** Craig Evan Pollack, Michelle E. Ross, Katrina Armstrong, Charles C. Branas, Karin V. Rhodes, Justin E. Bekelman, Alicia Wentz, Christian Stillson, Archana Radhakrishnan, Enny Oyeniran, David Grande

**Affiliations:** 1 Department of Medicine, Johns Hopkins School of Medicine and Bloomberg School of Public Health, Baltimore, Maryland, United States of America; 2 Department of Biostatistics and Epidemiology, Perelman School of Medicine, University of Pennsylvania, Philadelphia, Pennsylvania, United States of America; 3 Massachusetts General Hospital, Boston, Massachusetts, United States of America; 4 Office of Population Health Management, Northwell Health/Hofstra Medical School, Great Neck, New York, United States of America; 5 Department of Radiation Oncology, Abramson Cancer Center, Perelman School of Medicine, University of Pennsylvania, Philadelphia, Pennsylvania, United States of America; 6 Department of Epidemiology, Johns Hopkins Bloomberg School of Public Health, Baltimore, Maryland, United States of America; 7 Division of General Internal Medicine, Perelman School of Medicine, University of Pennsylvania, Philadelphia, Pennsylvania, United States of America; 8 Perelman School of Medicine, University of Pennsylvania, Philadelphia, Pennsylvania, United States of America; 9 Leonard Davis Institute of Health Economics, University of Pennsylvania, Philadelphia, Pennsylvania, United States of America; Taipei Medical University, TAIWAN

## Abstract

**Purpose:**

Prior work suggests that access to health care may influence the diagnosis and treatment of prostate cancer. Mystery-caller methods have been used previously to measure access to care for health services such as primary care, where patients’ self-initiate requests for care. We used a mystery-caller survey for specialized prostate cancer care to assess dimensions of access to prostate cancer care.

**Materials and Methods:**

We created an inventory of urology and radiation oncology practices in southeastern Pennsylvania. Using a ‘mystery caller’ approach, a research assistant posing as a medical office scheduler in a primary care office, attempted to make a new patient appointment on behalf of a referred patient. Linear regression was used to determine the association between time to next available appointment with practice and census tract characteristics.

**Results:**

We successfully obtained information on new patient appointments from 198 practices out of the 223 in the region (88.8%). Radiation oncology practices were more likely to accept Medicaid compared to urology practices (91.3% vs 36.4%) and had shorter mean wait times for new patient appointments (9.0 vs 12.8 days). We did not observe significant differences in wait times according to census tract characteristics including neighborhood socioeconomic status and the proportion of male African American residents.

**Conclusions:**

Mystery-caller methods that reflect real-world referral processes from primary care offices can be used to measure access to specialized cancer care. We observed significant differences in wait times and insurance acceptance between radiation oncology and urology practices.

## Introduction

Access to health care is a potentially important factor in men’s ability to undergo cancer treatment and may help explain observed differences in treatment by type of insurance, patient socioeconomic status, and race. [1, 2] However, little research has systematically examined dimensions of access beyond distance to care which may impact men’s ability and willingness to undergo treatment. We sought to explore the potential access experienced by men with localized prostate cancer to urology and radiation oncology clinics, including acceptance of new patients, length of time to an appointment, and types of insurance accepted. To do so, we employed a ‘mystery-caller’ approach in which a research assistant posed as a staff member at a patient’s primary care provider office and attempted to schedule a referral for a new patient appointment.

Mystery-caller approaches, also called ‘simulated customers’ or ‘secret shoppers’, were initially used to document racial discrimination in, for example, real estate, and have increasingly been used in health care to understand timeliness of outpatient appointments. [3–6] In these studies, research staff have typically posed as patients seeking to make a new patient appointment. To our knowledge, prior research has not used mystery-caller approaches to study access to prostate cancer care. Nor have these approaches been modified such that the research assistant poses as a physicians’ office assistant which may present a more accurate depiction of real world specialist referral workflows.

## Materials and Methods

The data come from the Philadelphia Area Prostate Cancer Access Study (P^2^ Access). This study focuses on how access to care influences racial disparities in prostate cancer treatment for men living in the greater Philadelphia area- a large, racially and ethnically diverse area containing approximately 5.3 million residents. This study was reviewed and approved by the University of Pennsylvania IRB (Protocol # 816864).

We generated a list of eligible urology and radiation oncology practices in the Philadelphia area and surrounding counties (25 total counties) using the National Provider Identifier database and a proprietary commercial database (SK&A). [7] From each source, we identified urologists and radiation oncologists, then sorted and de-duplicated by the address where they provide outpatient medical services. Clinics were geocoded using ArcGIS version 10.2 (ESRI, Redlands, CA) and linked to the characteristics of the census tracts in which they were located.

A research assistant posing as a scheduler from a primary care provider’s office called each practice to ask for the next available appointment for a new patient with private insurance (Independence Blue Cross) who had either an elevated Prostate Specific Antigen (PSA; for urology practices) or biopsy-confirmed prostate cancer (for radiation oncology practices). See S1 File for script. If asked by the specialists’ office staff, the simulated scheduler had information on the patient’s age (63 years old), date of birth, contact information (address, telephone number), and clinical characteristics (PSA level of 4.7 ng/ml for both clinical scenarios and, for radiation oncology clinics, a Gleason score of 6 and stage IIb). After determining the day and time of the next available new patient appointment, the simulated scheduler declined the offered appointment.

Practice respondents were further asked—“for future reference”- whether they accepted Medicare, Medicaid or self-pay (no insurance) patients; whether evening (>5pm) or weekend appointments were available; whether there was a nearby public transportation stop; and, if parking was available and whether it was free of charge. Mystery caller approaches preclude participants from providing informed consent prior to their participation. However, after each call, a debriefing letter was sent to the participating practice informing them of the call, its purpose, measures taken to preserve privacy, and providing contact information for both the investigator and the University of Pennsylvania IRB.

After summarizing the characteristics of the practices, we compared wait times at urology and radiation oncology practices using multivariable regression including practice characteristics (model 1) and additionally census tract characteristics (model 2).

## Results

We identified 223 unique, eligible practices, of which 151 (67.7%) were urology practices and 72 (32.3%) were radiation oncology practices (Table 1). Through this approach, we were able to obtain times for new patient appointments from 88.8% of practices called. The remaining practices (n = 25) required additional patient information (e.g., insurance identification number) in order to offer a specific appointment. Our ability to obtain an appointment time did not differ across urology vs. radiation oncology practices (89.4% vs. 87.5%) despite the different clinical context (initial evaluation for an elevated PSA vs. consultation for biopsy proven prostate cancer).

**Table 1:** Characteristics of practices in southeastern Pennsylvania and the surrounding counties.

	All practices N = 223	Urology practices N = 151	Radiation Oncologypractices N = 72
Accept Medicare	222 (99.55%)	150 (99.34%)	72 (100%)
Accept Medicaid	115 (54.25%)	52 (36.36%)	63 (91.3%)
Accept self-pay	199 (91.71%)	137 (91.33%)	62 (92.54%)
Average wait time in days (SD)^1^	11.60 (11.42)	12.82 (12.98%)	8.98 (6.36%)
Weekend appointments available^2^	5 (2.26%)	4 (2.68%)	1 (1.39%)
Evening appointments available^2^	20 (9.05%)	19 (12.75%)	1 (1.39%)
Parking available	221 (99.10%)	150 (99.34%)	71 (98.61%)
Near public transportation^3^	144 (64.57%)	93 (61.59%)	51 (70.83%)

^1^Wait time data missing from 25 practices (16 urology, 9 radiation oncology)

^2^Data missing from 2 practices (2 urology, 0 radiation oncology)

^3^Providers were asked if their office was near a public transit stop.

Only 5 practices (2.3%) had weekend appointments available, whereas 20 (9.1%) had evening appointments after 5pm. Approximately half of the practices accepted Medicaid (54.3%), with a higher proportion of radiation oncology practices accepting Medicaid compared to urology practices (91.3% versus 36.4%).

Among those that offered an appointment, the mean wait time to get an appointment was 11.6 days, with modestly shorter wait times found at radiation oncology practices compared to urology practices (9.0 for a patient diagnosed with cancer vs 12.8 days for a patient with an elevated PSA). In our adjusted model, radiation oncology practices had significantly shorter wait times than urology practices (-6.3 days, 95% CI -10.4, -2.3; P = 0.003, Table 2). In addition, practices that did not accept Medicaid had significantly shorter wait times than practices that accepted Medicaid (-3.9 days, 95% CI -7.7, 0.0; P = 0.05). We did not find significant differences in wait times based on other clinic characteristics or based on census tract characteristics.

**Table 2:** Multivariable linear regression model examining the association between wait time (in days) and practice characteristics.

	Model 1		Model 2^1^	
	Point estimate (days)^2^	95% Confidence Interval	Point estimate (days)^2^	95% Confidence Interval
**Intercept**	21	(4.6, 37.4)	6.1	(-35.8, 48.0)
**Accepts Medicaid**				
Yes	Ref	—	Ref	—
No	-3.9	(-7.7, 0.0)	-3.9	(-7.9, 0.1)
Not Sure	6.3	(-2.2, 14.8)	6.1	(-2.5, 14.7)
**Self Pay Allowed**				
Yes	Ref	—	Ref	—
No	1.1	(-5.1, 7.4)	1.9	(-4.5, 8.2)
Not Sure	-4	(-14.5, 6.4)	-0.9	(-11.7, 9.9)
**Near Public Transportation**				
Yes	Ref	—	Ref	—
No	3.4	(-1.9, 8.6)	3.3	(-2.4, 8.9)
Not Sure	-0.8	(-4.6, 2.9)	-1.2	(-5.1, 2.8)
**Parking Available**				
Yes	Ref	—	Ref	—
No	-5.7	(-21.5, 10.0)	-3.1	(-19.0, 12.9)
**Specialty type**				
Urology	Ref	—	Ref	—
Radiation Oncology	-6.3	(-10.4–2.3)	-6.8	(-11.0, -2.7)
**Weekend Appointments Available**				
No	Ref	—	Ref	—
Yes	-1.6	(-13.1, 9.8)	-2.9	(-14.5, 8.6)
**Proportion of male population with Medicaid**^3^			2.8	(-1.4, 7.0)
**Log of the Population Density**			-1.1	(-2.7, 0.4)
**Proportion of the male population that is African Americans**^3^^,^^4^			0.9	(-0.2, 2.0)
**Neighborhood Socioeconomic Status**^3^^,^^5^			2.1	(-1.4, 5.6)

^**1**^Model 2 is based on 193 unique providers. We exclude providers with missing wait time information (N = 25), missing weekend appointment status (N = 1), and missing census tract characteristics (N = 1).

^2^Point estimates represent differences in wait times in days.

^3^Point estimate correspond to a 10% point increase of the census tract characteristic.

^4^Proportion of the male population is restricted to men > 45 years old

^5^Neighborhood socioeconomic status ranging from 0 (low) to 100 (high) based on six ACS census tract variables: percent of adults older than 25 years with less than a high school education, percent of unemployed males, percent living in poverty, percent of households receiving public assistance, percent of female-headed households with children, and median household income.

## Discussion

Using a mystery caller approach to assess dimensions of access to prostate cancer care, we found slightly shorter wait times at radiation oncology practices compared to urology, albeit for different clinical scenarios (elevated PSA for urology and newly diagnosed prostate cancer for radiation oncology), and higher Medicaid acceptance at radiation oncology practices. Notably, relatively few clinics offered evening and weekend hours which may be especially important among working aged men.

This study has several limitations. We did not perform separate calls for patients with different types of insurance and did not address heterogeneity in wait times for providers within a given practice. Our focus on the Philadelphia region may limit its generalizability. Despite these limitations, the study demonstrates the potential for mystery caller approaches to document potential differences in access to care for urology and radiation oncology practices that may impact care delivery and racial disparities in prostate cancer treatment.

## Supporting Information

S1 File Mystery Caller Scripts for Urology and Radiation Oncology Practices. This document includes the two scripts used by a trained research assistant during calls to both urology (pages 1–4) and radiation oncology (pages 5–8) practices.
(DOC)
