# Supplementary material for: Using a Mystery-Caller Approach to Examine Access to Prostate Cancer Care in Philadelphia
Source: PLoS One. 2016 Oct 10;11(10):e0164411. doi: 10.1371/journal.pone.0164411 (PMC5056677; doi:10.1371/journal.pone.0164411)
Supplement: S1 File — This document includes the two scripts used by a trained research assistant during calls to both urology (pages 1–4) and radiation oncology (pages 5–8) practices. (DOC) [file pone.0164411.s001.doc]

**SCENERIO 1 (UROLOGY PROVIDERS)**

Provider Inventory “Scheduler” Script and Data Collection Form

1. Attempt Number**:** First  Second  Third
2. Date: Attempt 1:____/____/____ Attempt 2:____/____/_____ Attempt 3:____/____/_____
3. Start to End Time of phone call:

Attempt 1

____:____ am/pm *(start)*

____:____ am/pm *(end)*

Attempt 2

____:____ am/pm *(start)*

____:____ am/pm *(end)*

Attempt 3

____:____ am/pm *(start)*

____:____ am/pm *(end)*

1. Dial the provider’s telephone number: Phone Number Dialed: (___) ___________
2. Number of transfers until you reached the appointment scheduling line:

Attempt 1:______________ Attempt 2: ______________ Attempt 3: ______________

1. Number of people you spoke to during the call:

Attempt 1:______________ Attempt 2: ______________ Attempt 3: ______________

1. Answering Machine/ Voicemail. If a machine answers, or you are transferred to voicemail, DO NOT leave a message. *Write down any alternative contact number mentioned on the machine message/voicemail below if it is different than the telephone number you dialed in item 4.*

a**.** Attempt 1: Did you reach answering machine/voicemail?: Yes (proceed to item 8d*)* No (*proceed to item 9)*

b. Attempt 2: Did you reach answering machine/voicemail?: Yes (*proceed to item 8d)* No (*proceed to item 9)*

c. Attempt 3: Did you reach answering machine/voicemail?: Yes (*proceed to item 8d)* No (*proceed to item 9)*

1. Alternative Phone Number(s) if any: (___) ___________ **Use this number for subsequent attempts.*
2. If person answers:

**“Hello, I’m calling from Dr. Grande’s office. Is this where I can schedule an appointment for a new patient?**

Yes *(proceed)* No *(fill in item 7d if an alternative number is given and try again)*

**“Great. Dr. Grande has a patient with an elevated PSA level he would like to refer to [OFFICE/PROVIDER NAME]. May I schedule an appointment for this patient and ask a few questions about your practice for future reference?”**

1. Yes. *(proceed to item 9)*
2. If they aren’t scheduling new appointments for the moment *proceed to item 11.*
3. If they do not see or treat patients with elevated PSA levels *proceed to item 11.*
4. Attempt to schedule an appointment (*which you will later decline*) and ask the following questions:
5. **What is the next available appointment for this patient?** Date: ____/____/_____

Time: ____:____ am/pm

1. **Do you offer appointments on evenings (after 5pm) or weekends?** Evenings:  Yes  No

Weekends:  Yes  No

PATIENT INFORMATION

| **IF ASKED/TOLD, PLEASE MARK** | **YOUR RESPONSE** |
| --- | --- |
| *What is the patient’s name?* | Robert Leonard Davis |
| *What is the patient’s age/ date of birth?* | The patient is 63 years old. His date of birth is January 15^th^, 1949. |
| *What is the patient’s Social Security number?* | I don’t have that information available. |
| *What is the patient’s address?* | 2542 Washington Street  *City, County, and State (same as provider you are calling)  *Zip (change last 2 digits of center zip) |
| *What is the patient’s telephone number?* | *Purchased pre-paid cell phone number |
| *Does the patient have a cell phone number?* | No. We only have a home number on file. |
| *What is the patient’s marital status?* | I don’t have that information available. |
| *What is the patient’s race?* | I don’t have that information available. |
| *What is the patient’s primary complaint/ issue?* | An elevated PSA level. |
| *What is the patient’s PSA level?* | The patient has a PSA level of 4.7 ng/mL. |
| *When did the patient receive these PSA results?* | Recently. I believe about two weeks ago. |
| *We will need a copy of the patient’s test results.* | We will send the results later before the patient’s appointment. |
| *We already have this patient in our system.* | I’m sure this patient hasn’t visited before. |

PATIENT INSURANCE INFORMATION

| **IF ASKED/TOLD, PLEASE MARK** | **YOUR RESPONSE** |
| --- | --- |
| *What is the insurance company?* | Independence Blue Cross |
| *Is the patient the subscriber?* | Yes |
| *What is the insurance ID number?* | Our system is currently down, so I don’t have that information available at the moment. |
| *The insurance is effective from what date to what date?* | Our system is currently down, so I don’t have that information available at the moment. |
| *What is the patient’s subscriber ID?* | Our system is currently down, so I don’t have that information available at the moment. |
| *What is the group name?* | Our system is currently down, so I don’t have that information available at the moment. |
| *What is the group number?* | Our system is currently down, so I don’t have that information available at the moment. |

PRIMARY CARE PROVIDER INFORMATION

| **IF ASKED/TOLD, PLEASE MARK** | **YOUR RESPONSE** |
| --- | --- |
| *What is the primary care physician’s name?* | Dr. David Grande |
| *What is the primary care physician’s UPIN/NPI number?* | NPI: 1518978782 |
| *What is the primary care physician’s office address?* | 3701 Market Street, 7^th^ Floor, Suite 741  Philadelphia, PA 19104-4206 |
| *What is the primary care physician’s telephone number?* | **215-573-3804** |

EMERGENCY CONTACT INFORMATION

| **IF ASKED/TOLD, PLEASE MARK** | **YOUR RESPONSE** |
| --- | --- |
| *Can you provide me with the patient’s emergency contact information?* | Can Mr. Davis provide that information when he comes in for his appointment? |

1. *After asking the above questions/ If an appointment if offered:* **“Actually, I’m not sure that any of those times will work well for the patient. I think I’ll have to reconfirm with the patient and call back to schedule the appointment. Also,…”**
2. **“We like to keep our referral database updated and I don’t think we’ve updated your practice recently. Do you mind if I ask a few more questions about the practice for future referrals?”** *Check or write in the appropriate answer. If they want to transfer you, ask them to wait until you have finished asking questions. If they give you a name/ number to call for answers, record it in item 11j.*

| **IF ASKED/TOLD, PLEASE MARK** | **YOUR RESPONSE** |
| --- | --- |
| *I thought your system was down?* | We have our patient information and referral database in different systems. |

1. **Just to confirm, [OFFICE/PROVIDER NAME] is located at [OFFICE/PROVIDER BUSINESS ADDRESS] right?**

Yes

No, we are located at: **________________________________________________________________________**

**________________________________________________________________________**

1. **Does your office accept Medicare?**  Yes  No  Not Sure
2. **How about Medicaid or Medical Assistance?**  Yes  No  Not Sure
3. **And how about self-pay – can someone without health insurance pay cash?**  Yes  No  Not Sure
4. **Do you happen to know if there is a nearby public transportation stop?**  Yes  No  Not Sure
   1. **How long would it take to walk from the stop to where you are located?**

**____________________________________________________________**

1. **Do you offer parking for patients?**   Yes  No
   1. **Is there a charge?**  Yes  No  Not Sure
2. **How many urologists practice at this location? _________**
3. **What hospitals does the urologist(s) admit patients to?**

**_________________________________________________________________________________________________**

1. **Are there any radiation oncologists that also practice here?**  Yes  No
   1. **How many radiation oncologists practice here?** _________
2. *Transfer Names/ Phone Number(s) if any*: Name:________________ Number: (___) ___________
3. **“Thank you for your time. Have a nice day.”**

NOTES/COMMENTS ABOUT THE CALL:

Does the provider/office treat prostate cancer patients? ___Yes ___No

**SCENERIO 2 (RADIATION ONCOLOGY PROVIDERS)**

Provider Inventory “Scheduler” Script and Data Collection Form

1. Attempt Number**:** First  Second  Third
2. Date: Attempt 1:____/____/____ Attempt 2:____/____/_____ Attempt 3:____/____/_____
3. Start to End Time of phone call:

Attempt 1

____:____ am/pm *(start)*

____:____ am/pm *(end)*

Attempt 2

____:____ am/pm *(start)*

____:____ am/pm *(end)*

Attempt 3

____:____ am/pm *(start)*

____:____ am/pm *(end)*

1. Dial the provider’s telephone number: Phone Number Dialed: (___) ___________
2. Number of transfers until you reached the appointment scheduling line:

Attempt 1:______________ Attempt 2: ______________ Attempt 3: ______________

1. Number of people you spoke to during the call:

Attempt 1:______________ Attempt 2: ______________ Attempt 3: ______________

1. Answering Machine/ Voicemail. If a machine answers, or you are transferred to voicemail, DO NOT leave a message. *Write down any alternative contact number mentioned on the machine message/voicemail below if it is different than the telephone number you dialed in item 4.*

a**.** Attempt 1: Did you reach answering machine/voicemail?: Yes (proceed to item 8d*)* No (*proceed to item 9)*

b. Attempt 2: Did you reach answering machine/voicemail?: Yes (*proceed to item 8d)* No (*proceed to item 9)*

c. Attempt 3: Did you reach answering machine/voicemail?: Yes (*proceed to item 8d)* No (*proceed to item 9)*

1. Alternative Phone Number(s) if any: (___) ___________ **Use this number for subsequent attempts.*
2. If person answers:

**“Hello, I’m calling from Dr. Grande’s office. Is this where I can schedule an appointment for a new patient?**

Yes *(proceed)* No *(fill in item 7d if an alternative number is given and try again)*

**“Great. Dr. Grande has a patient newly diagnosed with prostate cancer he would like to refer to [OFFICE/PROVIDER NAME]. May I schedule an appointment for this patient and ask a few questions about your practice for future reference?”**

1. Yes. *(proceed to item 9)*
2. If they aren’t scheduling new appointments for the moment *proceed to item 11.*
3. If they do not see or treat prostate cancer patients: “**Thank you for your time.”** *(proceed to notes/comments at end )*
4. Attempt to schedule an appointment (*which you will later decline*) and ask the following questions:
5. **What is the next available appointment for this patient?** Date: ____/____/_____

Time: ____:____ am/pm

1. **Do you offer appointments on evenings (after 5pm) or weekends?** Evenings:  Yes  No

Weekends:  Yes  No

PATIENT INFORMATION

| **IF ASKED/TOLD, PLEASE MARK** | **YOUR RESPONSE** |
| --- | --- |
| *What is the patient’s name?* | Robert Leonard Davis |
| *What is the patient’s age/ date of birth?* | The patient is 63 years old. His date of birth is January 15^th^, 1949. |
| *What is the patient’s Social Security number?* | I don’t have that information available. |
| *What is the patient’s address?* | 2542 Washington Street  *City, County, and State (same as provider you are calling)  *Zip (change last 2 digits of center zip) |
| *What is the patient’s telephone number?* | *Purchased pre-paid cell phone number |
| *Does the patient have a cell phone number?* | No. We only have a home number on file. |
| *What is the patient’s marital status?* | I don’t have that information available. |
| *What is the patient’s race?* | I don’t have that information available. |
| *What is the patient’s primary complaint/ issue?* | Diagnosis of prostate cancer. |
| *What is the patient’s cancer stage?* | I believe it is Stage 2(B}. |
| *How was the patient diagnosed?* | Through a PSA test and biopsy. These tests resulted in a PSA level of 4.7 ng/mL and a Gleason score of 6. |
| *Has the patient had surgery?* | No. |
| *How long has the patient been diagnosed?* | About two weeks ago. |
| *Who diagnosed the patient?* | I’m not sure. |
| *We will need a copy of the patient’s test results.* | We will send the results later before the patient’s appointment. |
| *We already have this patient in our system.* | I’m sure this patient hasn’t visited before. |

PATIENT INSURANCE INFORMATION

| **IF ASKED/TOLD, PLEASE MARK** | **YOUR RESPONSE** |
| --- | --- |
| *What is the insurance company?* | Independence Blue Cross |
| *Is the patient the subscriber?* | Yes |
| *What is the insurance ID number?* | Our system is currently down, so I don’t have that information available at the moment. |
| *The insurance is effective from what date to what date?* | Our system is currently down, so I don’t have that information available at the moment. |
| *What is the patient’s subscriber ID?* | Our system is currently down, so I don’t have that information available at the moment. |
| *What is the group name?* | Our system is currently down, so I don’t have that information available at the moment. |
| *What is the group number?* | Our system is currently down, so I don’t have that information available at the moment. |

PRIMARY CARE PROVIDER INFORMATION

| **IF ASKED/TOLD, PLEASE MARK** | **YOUR RESPONSE** |
| --- | --- |
| *What is the primary care physician’s name?* | Dr. David Grande |
| *What is the primary care physician’s UPIN/NPI number?* | NPI: 1518978782 |
| *What is the primary care physician’s office address?* | 3701 Market Street, 7^th^ Floor, Suite 741 Philadelphia, PA 19104-4206 |
| *What is the primary care physician’s telephone number?* | **215-573-3804** |

EMERGENCY CONTACT INFORMATION

| **IF ASKED/TOLD, PLEASE MARK** | **YOUR RESPONSE** |
| --- | --- |
| *Can you provide me with the patient’s emergency contact information?* | Can Mr. Davis provide that information when he comes in for his appointment? |

1. *After asking the above questions/ If an appointment if offered:* **“Actually, I’m not sure that any of those times will work well for the patient. I think I’ll have to reconfirm with the patient and call back to schedule the appointment. Also,…”**
2. **“We like to keep our referral database updated and I don’t think we’ve updated your practice recently. Do you mind if I ask a few more questions about the practice?”** *Check or write in the appropriate answer. If they want to transfer you, ask them to wait until you have finished asking questions. If they give you a name/ number to call for answers, record it in item 11j.*

| **IF ASKED/TOLD, PLEASE MARK** | **YOUR RESPONSE** |
| --- | --- |
| *I thought your system was down?* | We have our patient information and referral database in different systems. |

1. **Just to confirm, [OFFICE/PROVIDER NAME] is located at [OFFICE/PROVIDER BUSINESS ADDRESS] right?**

Yes

No, we are located at: **________________________________________________________________________**

**________________________________________________________________________**

1. **Does your office accept Medicare?**  Yes  No  Not Sure
2. **How about Medicaid or Medical Assistance?**  Yes  No  Not Sure
3. **And how about self-pay – can someone without health insurance pay cash?**  Yes  No  Not Sure
4. **Do you happen to know if there is a nearby public transportation stop?**  Yes  No  Not Sure
   1. **How long would it take to walk from the stop to where you are located?**

**____________________________________________________________**

1. **Do you offer parking for patients?**   Yes  No
   1. **Is there a charge?**  Yes  No  Not Sure
2. **How many radiation oncologists practice at this location? _________**
3. **What hospitals does the radiation oncologist(s) admit patients to?**

**_________________________________________________________________________________________________**

1. **Are there any urologists that also practice here?**  Yes  No
   1. **How many urologists practice here? _________**
2. *Transfer Names/ Phone Number(s) if any*: Name:________________ Number: (___) ___________
3. **“Thank you for your time. Have a nice day.”**

NOTES/COMMENTS ABOUT THE CALL:

Does the provider/office treat prostate cancer patients? ___Yes ___No
